# When the Professional Turns Personal: Healthcare Providers and Their Own Advance Care Planning During the Pandemic

**DOI:** 10.1093/geroni/igab046.513

**Published:** 2021-12-17

**Authors:** Brian Carpenter

**Affiliations:** Washington University in St. Louis, Saint Louis, Missouri, United States

## Abstract

As healthcare professionals counseled patients and care partners during the pandemic about treatment options, clinical probabilities, and preparations for death, they had opportunities to reflect on how they themselves would want to be treated if they fell ill. We conducted a survey with healthcare professionals who were caring for patients during the pandemic and asked their work had affected their own advance care planning. Based on their clinical observations, 28% revised their personal interest in life-prolonging medical interventions. Substantial proportions had initiated conversations with partners (45%), parents (46%), and their primary care physician (29%) about their medical preferences. Similarly high percentages had encouraged family members and friends to update or complete their advance care plans, and 26% intended to initiate planning in the near future. Interest in advance care planning is high among healthcare professionals, who may benefit from tailored resources that acknowledge their clinical experience.

